# Pocketable Biosensor Based on Quartz-Crystal Microbalance and Its Application to DNA Detection

**DOI:** 10.3390/s23010281

**Published:** 2022-12-27

**Authors:** Hiroshi Yoshimine, Kai Sasaki, Hiroyuki Furusawa

**Affiliations:** 1 Graduate School of Science and Engineering, Yamagata University, Yonezawa 992-8510, Japan; 2 Institute for the Promotion of General Graduate Education (IPGE), Yamagata University, Yonezawa 992-8510, Japan

**Keywords:** quartz-crystal microbalance, biosensor, IC card size, DNA hybridization, single-base mismatch detection

## Abstract

Quartz-crystal microbalance (QCM) is a technique that can measure nanogram-order masses. When a receptor is immobilized on the sensor surface of a QCM device, the device can detect chemical molecules captured by the mass change. Although QCM devices have been applied to biosensors that detect biomolecules without labels for biomolecular interaction analysis, most highly sensitive QCM devices are benchtop devices. We considered the fabrication of an IC card-sized QCM device that is both portable and battery-powered. Its miniaturization was achieved by repurposing electronic components and film batteries from smartphones and wearable devices. To demonstrate the applicability of the card-sized QCM device as a biosensor, DNA-detection experiments were performed. The card-sized QCM device could detect specific 10-mer DNA chains while discerning single-base differences with a sensitivity similar to that of a conventional benchtop device. The card-sized QCM device can be used in laboratories and in various other fields as a mass sensor.

## 1. Introduction

Observing biomolecular interactions with proteins (peptides), nucleic acids (DNA and RNA), saccharides, and other bio-related molecules is required to elucidate biological mechanisms in primary research fields. However, labeling these biomolecules can often alter their natural behavior. Therefore, devices for biomolecular interaction analysis (BIA) have been developed to achieve the label-free detection of biomolecules based on various principles, including optical sensing, isothermal titration calorimetry, electrochemical sensing, and acoustic sensing [1]. Optical surface plasmon resonance (SPR) sensors are widely used in BIA [2]. BIA devices are often made for benchtops and intended for use in laboratories owing to the high demand for BIA in basic research [1].

Recently, the development of handheld biochemical sensors for healthcare and environmental monitoring has attracted considerable attention [3]. For example, healthcare devices developed for point-of-care are designed to detect biomarkers, often peptides or RNA, at home [4,5]. Many handheld devices use optical technology due to the evolution of accessories such as cameras and light sensors [3]. However, each principle employed in BIA sensors has advantages and disadvantages [6,7]. Therefore, in the field of handheld devices, it is desirable to have a complementary system that is based on different principles.

A quartz-crystal microbalance (QCM) is an acoustic sensor [8,9]. It utilizes a quartz-crystal plate as an oscillator. When the plate oscillates regularly via an oscillation circuit, the adsorption of a substance on the quartz plate causes a decrease in frequency according to its mass [10]. Therefore, QCM technologies are used in biosensors that take mass measurements [11,12]. Using a quartz plate on which a receptor is immobilized as a sensor surface, a QCM device could detect captured chemical molecules by mass change. The frequency change (∆*F*) of the plate could be applied to the calculation of the change in mass (∆*m*) using Equation (1):

∆*m* = −*K*∆*F*, (1)

where ∆*m* is the mass change [in ng cm^−2^], ∆*F* is the measured frequency change [in Hz]. K is a proportionality constant [in ng cm^−2^ Hz^−1^] that depends on the fundamental frequency (*F*_0_) with the overtone number (*n*) of the quartz-crystal oscillator and the measurement phase, such as in air or water [13]. In the case of *F*_0_ = 27 MHz (*n* = 1) and measurements in the air phase, the *K* value has been identified as 0.62 ± 0.02 ng cm^−2^ Hz^−1^ [13]. Thus, when measuring ∆*F* in the range of 1–10,000 Hz, the QCM can measure masses on the order of nanograms.

**Figure 1:**
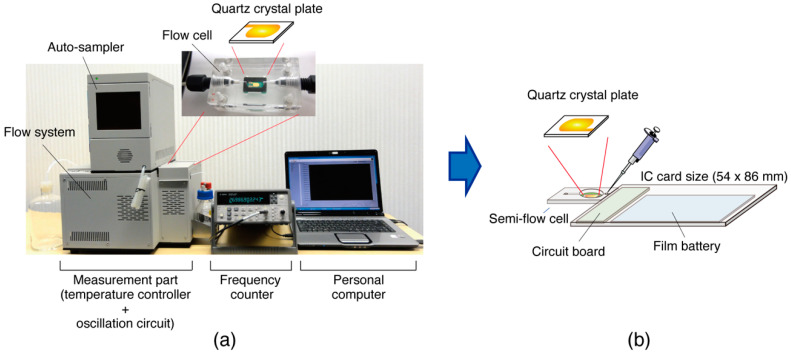
Schematic illustrations of (**a**) a previously fabricated 27 MHz QCM device designed to achieve a low noise level [14] and (**b**) a pocketable 27 MHz QCM device designed to prioritize portability.

Previously, we fabricated a 27 MHz QCM device for BIA that was equipped with an auto-sampler, flow system, and temperature controller to achieve a low noise level of ± 0.1 Hz [14]. Using the low-noise QCM device, we observed DNA hybridization and enzymatic reactions on double-stranded DNA as nanogram-order mass changes [14,15]. The low-noise QCM device allowed us to kinetically analyze the enzymatic behavior. However, the device was designed as a benchtop device for use in the laboratory, using a benchtop frequency counter to measure the vibration of the oscillating quartz plate with high resolution (0.1–0.01 Hz) (Figure 1a)—That is, the resolution of the frequency counter (<0.1 Hz) needed to suit the oscillation stability (±0.1 Hz) of the quartz plate. Most highly sensitive QCM devices require a benchtop frequency counter to measure crystal oscillations with an accuracy of 1 Hz.

To extend the use of QCM devices beyond the laboratory, we considered the fabrication of a handheld QCM device while maintaining the ability to observe biomolecular interactions. Unlike the resources used in previous development environments, we repurposed small, inexpensive, high-performance electronic components from smartphones and wearable devices that were of millimeter-order with respect to size and less than 1.0 mm thick. Film batteries have also been used. This study investigated the fabrication of a compact and portable QCM device with the size of an IC card (Figure 1b). The card-sized QCM device was then used in DNA detection experiments to demonstrate its applicability. 

## 2. Materials and Methods

### 2.1. Sensor Chip

**Figure 2:**
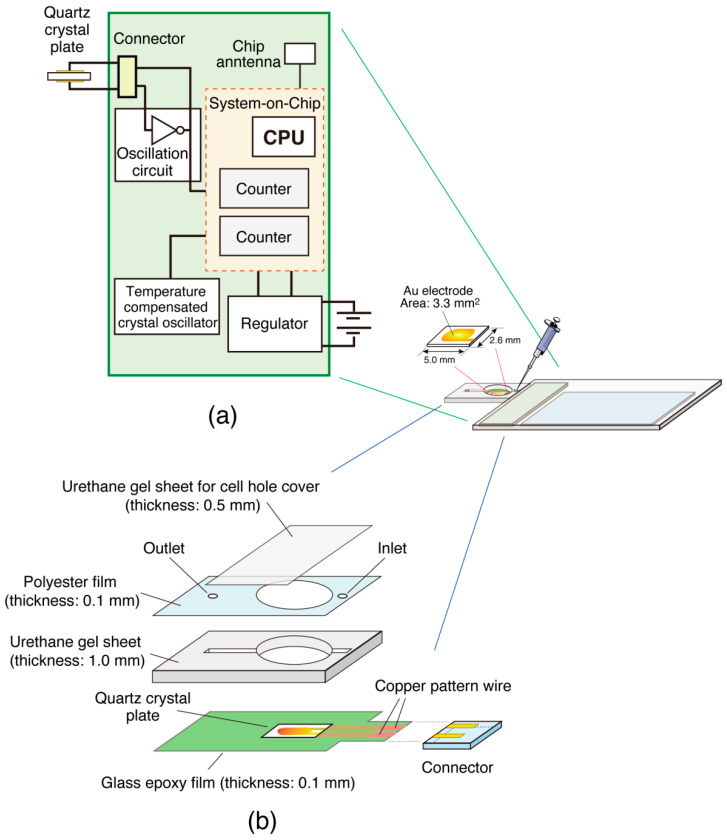
Schematic illustrations of (**a**) the circuit board of the card-sized QCM device and (**b**) the sensor chip with a quartz-crystal oscillator combined with a semiflow cell.

Figure 2 shows the card-sized QCM device. A rectangular 27 MHz AT-cut quartz crystal plate was custom-made by Piezo Parts Co., Ltd. (Hachioji, Japan). The quartz plate (size: (*L*) 5.0 × (*W*) 2.6 mm × (*T*) 60 µm) was equipped with an Au electrode (3.3 mm^2^) to function as an oscillator. The oscillator was attached to a glass epoxy film (thickness: 0.1 mm) using an elastic sealant around the quartz plate edge to ensure liquid contact on only one side of the quartz plate, as previously described [14]. The sensitivity of this oscillator as a mass sensor, when converted per electrode (3.3 mm^2^) from 0.62 ng cm^−2^ Hz^−1^, was estimated to be equivalent to a 1 Hz decrease with an increase in mass of 20 pg.

### 2.2. Device Design

A circuit board was designed to include the oscillation circuit and frequency counter functions required for the QCM devices (Figure 2a). A connector designed for flexible printed circuits with a height of 0.85 mm (6293 connector; Kyocera Co., Kyoto, Japan) was used to connect the glass epoxy film on which the oscillator was attached to an oscillation circuit on the circuit board. As previously reported, a Colpitts-type oscillation circuit was used to drive the oscillator [14]. A system-on-chip (SoC) (nRF52832; Nordic Semiconductor, Trondheim, Norway) with a size of (*L*) 3.0 × (*W*) 3.2 × (*T*) < 0.4 mm, which has a counter function for applications such as sensors for sports and fitness, was used to count the number of oscillations of the quartz-crystal oscillator. A highly stable 32 MHz oscillator (temperature-compensated XTAL oscillator: TCXO) with a size of (*L*) 1.6 × (*W*) 1.2 × (*T*) 0.45 mm for smartphones and wireless communication devices (TG1612SLN; Seiko Epson Co., Suwa, Japan) was used as the time base for accurate time measurement. These parts and other electronic components were placed on a printed wiring board and manufactured into printed circuit boards.

A film-type lithium-ion battery manufactured by Panasonic Co. (Osaka, Japan) (size: (*L*) 40.0 × (*W*) 65.0 × (*T*) 0.55 mm, battery capacity: 60 mAh, output voltage: 3.8 V) or by NGK Insulators, Ltd. (Aichi, Japan) (size: (*L*) 38.0 × (*W*) 27.0 × (*T*) 0.45 mm, battery capacity: 27 mAh, output voltage: 3.8 V) was used through a regulator as a drive power supply for the oscillator, the SoC, and the other electronic components on the circuit board. When using NGK batteries, the two film batteries were connected in parallel to ensure a battery capacity greater than 50 mAh. A chip antenna was mounted to transmit the measurement data to a smartphone or tablet via Bluetooth communication; however, this antenna was not used in this experiment. Measurement data were acquired from the device via a USB cable using a J-Link (Segger Microcontroller GmbH, Monheim, Germany) debug probe.

### 2.3. Semiflow Cell

As shown in Figure 2b, a semiflow cell was fabricated from a combination of three sheets on the sensor chip, as described in Section 2.1. A hole with a radius of 4 mm was cut from the urethane gel sheet (thickness: 1.0 mm) to serve as the measurement cell (volume: 50 µL). A sample injection channel was created by creating grooves on both sides of the measurement cell and covering it with a polyester film (thickness: 0.1 mm) with small holes at the inlet and outlet. During the interaction experiments, the measurement cell hole was covered with a urethane gel sheet (thickness: 0.5 mm).

### 2.4. DNA Detection

#### 2.4.1. Materials

Single-stranded (10-mer) and biotinylated oligonucleotides (10-mer) were purchased from Eurofins Genomics, K.K. (Tokyo, Japan). The chemical reagents, 3,3-dithiodipropionic acid (Nacalai Tesque, Kyoto, Japan), 1-Ethyl-3-[3-(dimethylamino)propyl]carbodiimide (EDC) (Dojindo Laboratories, Kumamoto, Japan), *N*-Hydroxysuccinimide (NHS) (Wako Pure Chemical Industries, Ltd., Osaka, Japan), and NeutrAvidin (Thermo Fisher Scientific Inc., Tokyo, Japan) were used for experiments with DNA. NaCl (Wako Pure Chemical Industries, Ltd., Osaka, Japan), Tris(hydroxymethyl)aminomethane (Wako Pure Chemical Industries, Ltd., Osaka, Japan), 2-[4-(2-Hydroxyethyl)-1-piperazinyl]ethanesulfonic acid (HEPES) (Dojindo Laboratories, Kumamoto, Japan), and Ethylenediamine-*N*,*N*,*N’*,*N’*-tetraacetic acid (EDTA) (Dojindo Laboratories, Kumamoto, Japan) were used to prepare the buffer solutions. Ultrapure water (Milli-Q) was used in all the experiments.

#### 2.4.2. Immobilization of Probe DNA

Probe DNA (probe DNA: biotin-5′-AGCTGGGGAA-3′) was immobilized on the sensor surface to capture the target DNA, as described elsewhere [16,17]. Briefly, the Au electrode of a sensor chip fabricating a semiflow cell was cleaned and subsequently modified with 3,3-dithiodipropionic acid through an Au-S interaction. A mixture of EDC and NHS was drop-cast onto the modified Au electrode to activate the carboxylic acids as *N*-hydroxysuccinimidyl esters. After rinsing the sensor surface with Milli-Q water, the measurement cell hole was covered with a gel sheet. The sensor chip with a semiflow cell was connected to the card-sized QCM device via a connector, and the measurement cell was filled with HEPES buffer solution (10 mM HEPES-NaOH, pH 7.9, 0.2 M NaCl) by injecting 100 µL of the buffer solution through the inlet hole with a micropipette. The quartz-plated oscillator on the sensor chip was oscillated using an oscillation circuit. To immobilize NeutrAvidin (NAv) as a biotin-binding protein on the sensor surface, 100 µL of NAv in the HEPES buffer at a concentration of 60 µg/mL was injected into the measurement cell and incubated for 1 h at 21 °C room temperature. After incubation, the flow cell was washed several times with 100 µL of Tris buffer solution (10 mM Tris-HCl, pH 8.0, 1 mM EDTA, 0.15 M NaCl). The biotinylated probe DNA solution of Tris buffer at a concentration of 200 nM was allowed to flow into the measurement cell with a micropipette to immobilize the probe DNA. The flow of DNA solution was repeated until a predetermined frequency (−150 Hz) was reached. Finally, the solution in the measurement cell was replaced with fresh Tris buffer solution, which filled the cell.

#### 2.4.3. Observation of Target-DNA Capture

The QCM sensor chip, on which the probe DNA was immobilized, was oscillated in Tris buffer solution at 21 °C with a card-sized QCM device. After confirming stable sensor chip oscillation, 100 µL of the DNA solution in Tris buffer at a concentration of 200 nM of target DNA (target-DNA:5′-TTCCCCAGCT-3′) or mismatch DNA (mismatch-DNA:5′-TTCCCAAGCT-3′) was injected into the measurement cell from the inlet hole using a micropipette. The frequency changes over time were then monitored. After the DNA-binding experiment, the sensor surface was rinsed with a 10 mM NaOH solution to remove the captured DNA, and the sensor was repeatedly used.

## 3. Results and Discussion

### 3.1. Fabrication of the Card-Sized QCM Device

**Figure 3:**
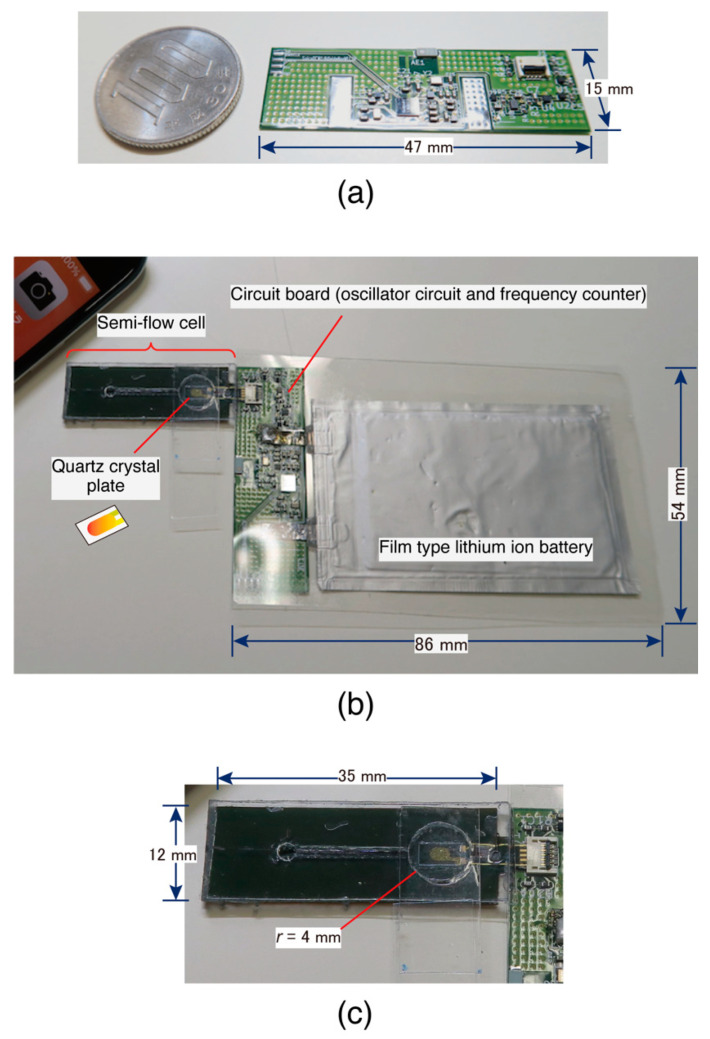
Photos of the card-sized QCM device; (**a**) the circuit board, (**b**) the overall view of the connected semiflow cell, and (**c**) the enlarged semiflow cell.

Figure 3 shows the fabricated QCM device used in this study. As shown in Figure 3a, the circuit board was manufactured using a polyimide substrate of size (*L*) 47.0 × (*W*) 15.0 × (*T*) 0.4 mm. The entire card-sized device is shown in Figure 3b. A circuit board with a film-type lithium-ion battery can be fabricated with a length and width that fits within an international standard IC card ((*L*) 53.98 × (*W*) 85.60 mm). The largest portion (connector part) of the overall thickness was 1.25 mm, which was larger than the thickness of an IC card (0.76 mm). We have confirmed that a circuit board can be produced using a glass epoxy substrate with a thickness of 0.15 mm on other manufacturing opportunities. An overall thickness of 1.0 mm was achievable in our study.

The semiflow cell shown in Figure 3c was handmade in our laboratory. A micropipette was used to inject 100 µL of the solution from the inlet, and a Kimwipe was used to absorb the solution from the outlet. This semiflow operation allowed us to displace the solution in the measurement cell (volume: 50 µL). To avoid increasing the internal pressure in the measurement cell during the injection of the solution from the inlet, a wide flow channel was provided on the outlet side to facilitate solution discharge. However, the wide channel caused the evaporation of the solution from the outlet. Therefore, the outlet side’s flow path was lengthened so that evaporation would not affect the solution in the measurement cell.

### 3.2. Resolution of Frequency Counter

The oscillation stability of the rectangular 27 MHz quartz-crystal oscillator prepared in this study was expected to be less than ±0.1 Hz because this oscillator exhibited a similar *Q* value to that of our oscillator, which achieved ±0.05 Hz in a previous study [14]. Therefore, we targeted approximately 1 Hz as the resolution of the frequency counter in this card-sized QCM device.

**Figure 4:**
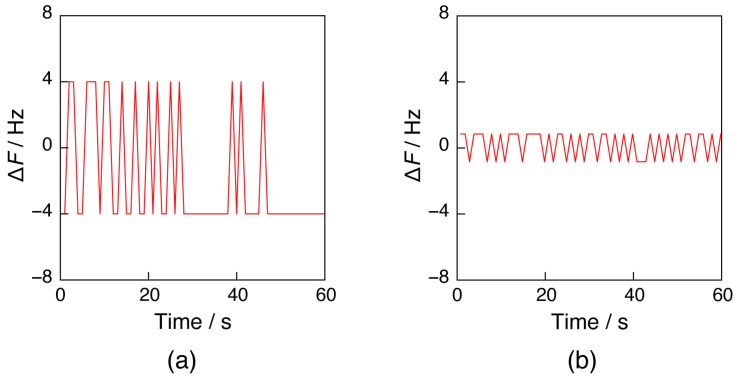
Frequency plots of a 27 MHz quartz oscillator on the sensor chip over time as measured by the card-sized frequency counter using (**a**) a direct method and (**b**) a reciprocal method.

To realize a MHz frequency counter with a single semiconductor chip, we repurposed an SoC with counter functions that were applied to wearable devices. The SoC had five counters available, which could also be used as a timer to provide a reference time. The entire SoC could be driven based on a highly stable 32 MHz TCXO. The counter in the SoC was driven at 16 MHz. However, the upper-frequency limits to which the timer and counter could adapt were 16 and 4 MHz, respectively. Therefore, the frequency of a 27 MHz oscillator on a sensor chip was reduced by three pre-scalers to 1/8 of the frequency (3.375 MHz), then counted for 1 s of the reference time by the SoC’s counter, and then back to 8 times (27 MHz) to obtain the frequency *F* (Figure 4a). As a result, the oscillation circuit and frequency counter in the card-sized QCM could drive the 27 MHz quartz-crystal oscillator and count the frequency. Although the resolution of the counter on the SoC was 1 Hz, the resulting resolution was 8 Hz. A reciprocal method was used to improve resolution. Thus, the 1/8 frequency of a 27 MHz oscillator was counted until it reached a predetermined number (*C* = 3.25 M) below 3.375 M, and then the frequency *F* [Hz] was calculated from Equation (2) based on the time *T* [s] that the timer was run at 16 MHz:

*F* = (*T*/*C*)^−1^. (2)

As shown in Figure 4b, the resolution improved to 1.7 Hz because of the 16 M/3.375 M-fold improvement from 8 Hz.

### 3.3. Confirmation of QCM Oscillation in Air and Water

**Figure 5:**
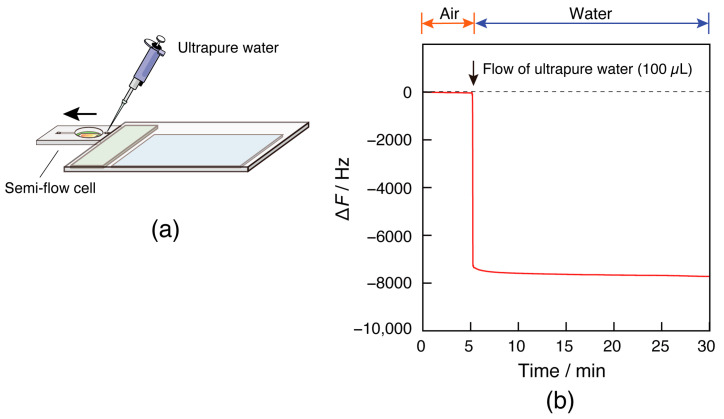
(**a**) Illustration of the water injection experiment on the card-sized QCM device and (**b**) frequency change in response to the injection of ultrapure water at 21 °C.

The oscillation of the 27 MHz quartz oscillator connected to the card-sized QCM device was confirmed in both air and water (Figure 5). While monitoring the frequency of the sensor chip, ultrapure water flowed into an empty semiflow cell (Figure 5a). The frequency decreased by approximately 8000 Hz due to the water contact on the sensor surface (Figure 5b). This value is in close agreement with ∆*F* = −8961 Hz obtained from Kanazawa–Gordon’s equation, which is reported as the theoretical frequency change of a QCM in solution [18]. This indicates that the card-sized QCM sensor can function properly as a QCM. When the oscillator on the sensor chip oscillated in water, the drive time of the oscillator was approximately 2 h, which is considered sufficient as the measurement time for a typical biosensor.

### 3.4. DNA Detection Experiment

#### 3.4.1. Monitoring of Protein and DNA Immobilization Processes

**Figure 6:**
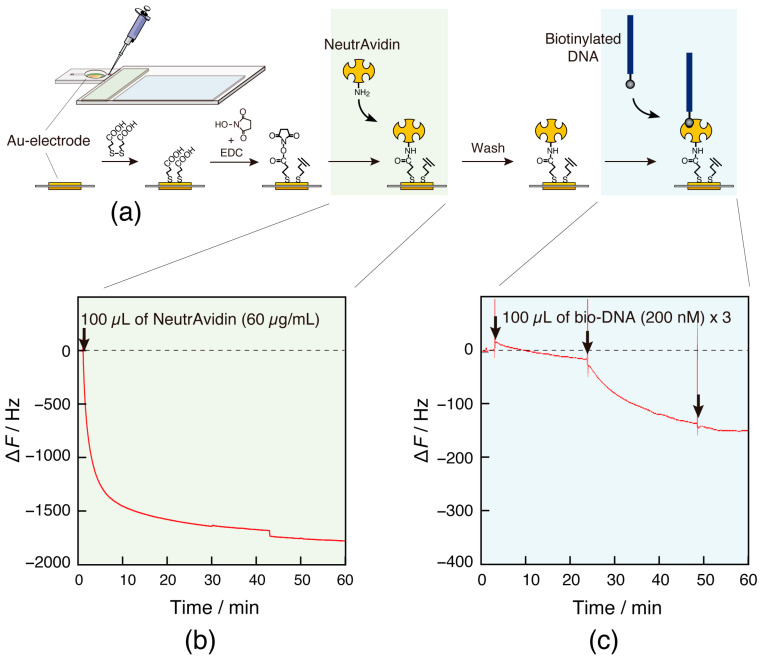
(**a**) Schematic illustration of DNA immobilization process on the QCM sensor surface. Time courses of a frequency change (∆*F*), responding to bindings of (**b**) NeutrAvidin to the activated carbonic acids and (**c**) biotinylated DNA (10-mer) to the NeutrAvidin on the QCM sensor surface in each buffer solution: 10 mM HEPES-NaOH (pH 7.9), 0.2 M NaCl for the NeutrAvidin binding and 10 mM Tris-HCl (pH 8.0), 1 mM EDTA, 0.15 M NaCl for biotinylated DNA binding at 21 °C.

A DNA detection experiment was carried out to confirm that the card-sized QCM device could be applied as a biosensor. First, the probe DNA (10-mer) was immobilized on the sensor surface as a receptor to capture the target DNA (10-mer) according to the avidin-biotin linkage method (Figure 6a) [17]. In this process, the immobilization behavior of NeutrAvidin (60 kDa) on the sensor surface was monitored over time (Figure 6b). Injection of the NeutrAvidin solution into the measurement cell resulted in an approximate 1800 Hz decrease in frequency (a mass increase). This is consistent with the results obtained from the conventional benchtop QCM devices. Thus, the protein-binding behavior could be observed with a sufficient sensing sensitivity of the card-sized QCM device. Subsequently, immobilization of the probe DNA (3.5 kDa) was observed on the card-sized QCM device (Figure 6c), and a frequency decrease (−145 Hz) was observed. When injecting, spike noise was observed owing to increased internal pressure in the measurement cell. Because the frequency quickly returned to its original level, it was determined that this did not affect the signal change.

#### 3.4.2. DNA Detection

**Figure 7:**
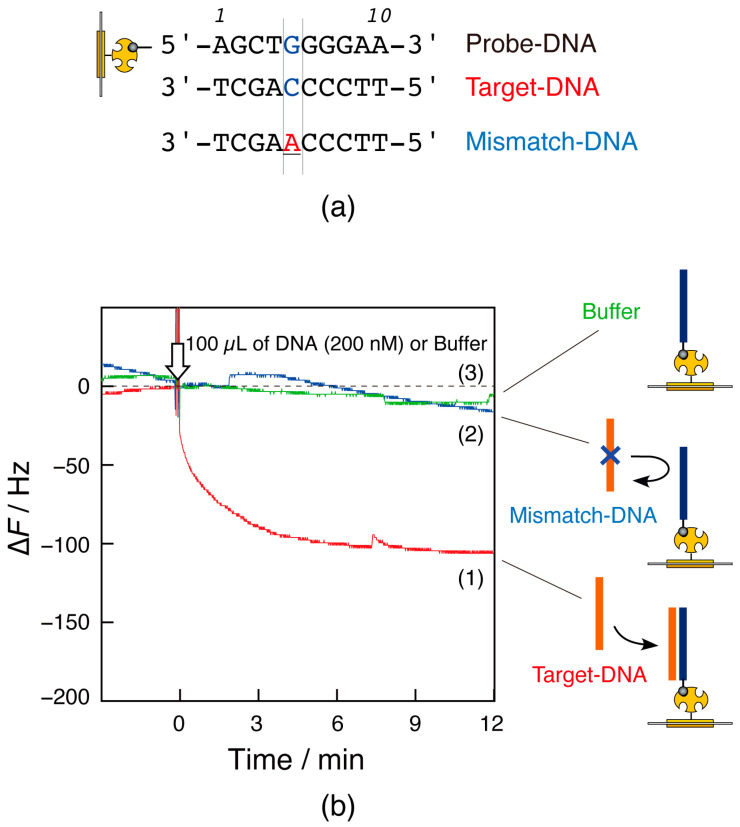
(**a**) DNA sequences used in this experiment; (**b**) time courses of frequency changes (∆*F*), responding to the injection of (1) Target-DNA, (2) Mismatch-DNA, and (3) the buffer solutions onto the semiflow cell of the card-sized QCM in the experimental condition: 10mM Tris-HCl (pH 8.0), 1 mM EDTA, 0.15 M NaCl at 21 °C.

The DNA to be detected was a 10-mer target-DNA (3.0 kDa) with a complementary sequence to the probe DNA (Figure 7a). Mismatch-DNA (3.0 kDa), in which one base in the center differed from the target-DNA sequence, was used for comparison. When the target-DNA solution was injected into the measurement cell, a 100 Hz decrease in frequency was observed (Figure 7b, curve 1). However, injection of the mismatch-DNA solution caused little to no decrease in frequency (curve 2). Because some signal drift (10 Hz/10 min) was observed only in the case of the buffer solution (curve 3), the change in curve 2 would not be due to a change in mass. Similar results were obtained in 11 DNA detection experiments. These results indicate that the card-sized QCM device could detect the target DNA’s capture by the probe DNA by distinguishing a single base difference. 

An observed frequency change of −100 Hz was discernible in the signal-to-noise ratio. This change corresponds to a 2.0 ng mass change on the electrode. This shows that the card-sized QCM device can adequately measure mass changes on the order of nanograms. This detection sensitivity was comparable to a conventional benchtop device [17]. However, compared to the conventional device, the binding rate was slower in the card-sized QCM because the solution in the measurement cell was not stirred. It should be noted that some signal drift was observed. This could be attributed to using both the device and the sensor chip in a room-temperature environment without temperature control or electrical shielding.

## 4. Conclusions

We investigated the development of a pocketable QCM device capable of measuring nanogram-order mass. An IC card-sized QCM device that can operate for several hours can be fabricated using a low-energy SoC and powerful film-type lithium-ion battery. Although the card-sized QCM device was not equipped with essential parts such as a temperature controller and stirring device to stabilize the crystal oscillator, the QCM device could be applied as a biosensor capable of detecting 10-mer DNA strands. This would allow the QCM method to be used as a biosensor in or outside the laboratory and as an embedded detector in various devices, such as wearable devices.

## 5. Patents

A patent application is pending with Yamagata University and Piezo Parts Co., Ltd.

## Data Availability

Not applicable.
